# The effect of calcitriol on the development and implantation capacity of embryos from hyper-stimulated mice

**DOI:** 10.3389/fimmu.2023.1200704

**Published:** 2023-07-21

**Authors:** Zoltán Bognár, Timea Judith Csabai- Tanics, Éva Görgey, Éva Mikó, Zoltán Horváth-Szalai, Júlia Szekeres-Barthó

**Affiliations:** ^1^ Department of Medical Biology and Central Electron Microscopy Laboratory, Medical School, Pecs University, Pecs, Hungary; ^2^ Szentágothai János Research Center, Pecs, Hungary; ^3^ National Laboratory on Human Reproduction, Pecs University, Pecs, Hungary; ^4^ MTA - PTE Human Reproduction Research Group, Pecs, Hungary; ^5^ Department of Medical Microbiology and Immunology, Medical School, Pecs University, Pecs, Hungary; ^6^ Department of Laboratory Medicine, Medical School, Pecs University, Pecs, Hungary

**Keywords:** Vitamin D, fertilization, embryo development, implantation, PIBF

## Abstract

The evidence concerning the role of vitamin D (VD) in reproduction is still inconclusive. Calcitriol was given to superovulated female mice at the time of FSH injection (Group A), or at day 0.5 of pregnancy (Group B). The retrieved and cultured embryos were transferred to the uteri of pseudopregnant females. Ten animals from each group conceived naturally, and at day 7.5 of pregnancy, the implantation sites were counted. Serum hormone concentrations were determined by ELISA. The expression of CD70, PD-L1, OX-40L, and PIBF on extracellular vesicles (EVs) was tested by flow cytometry. Calcitriol treatment did not alter serum oestradiol concentrations, while 25(OH) D levels significantly decreased in both treated groups. Progesterone concentrations were significantly higher in group A and lower in group B than in the controls. On EVs produced by group B embryos PIBF, CD70, and OX-40L expression were significantly lower, while that of PD-L1 was significantly higher than that of controls. Calcitriol treatment decreased the fertilization rate in group A, and the blastulation rate of cultured embryos in group B, while the implantation capacity of the embryos was not affected, suggesting that depending on the time of administration, VD has an adverse effect on oocyte maturation and embryo development, but not on the implantation rates.

## Introduction

While the role of vitamin D in bone metabolism is well established, there is no consensus concerning its effects on the immune system and reproduction.

Vitamin D originally described as a vitamin, is now considered a hormone, acting on its receptor that belongs to the superfamily of nuclear steroid receptors. Vitamin D receptors (VDRs) are present at various sites of the body, among others in granulosa cells and endometrial cells (1) as well as on immune cells (2, 3), suggesting that both of these systems might be affected by this hormone.

During conception and normal pregnancy, women undergo immunological changes consistent with the weakening of Th1 responses and strengthening of Th2 responses (4). Conversely, the activation of some forms of maternal cellular immune functions is potentially hazardous for foetal development (5). Progesterone –via its downstream mediator, PIBF- plays a role in establishing the Th2 dominant cytokine production during pregnancy (6).

Compared to non-pregnant women, there is a significant increase in 1,25(OH)2D concentrations during pregnancy, followed by a rapid decline after delivery (7). Although vitamin D requirements are not characteristically altered during gestation (8), low maternal vitamin D concentrations might affect the outcome of pregnancy. Vitamin D levels are generally determined by measuring 25(OH) vitamin D in the serum. 25(OH) vitamin D levels below 12 ng/mL (30 nmol/L) are considered deficient, and, those above 30 ng/mL (75 nmol/L) sufficient (7). Vitamin D levels in the serum and follicular fluid are in the same range (9–13). Normal serum concentrations might vary by race and geographical region (8, 13, 14).

Several studies revealed a relationship between 25(OH)D serum levels and reproductive parameters in humans (9, 15–17) and rodents (18), while other findings suggest that lower than normal vitamin D levels have no adverse effect on the success of IVF-ET (10–14, 19, 20).

Most studies investigate the relationship between the existing serum vitamin D levels and reproductive success, while only a few investigate the effect of vitamin D supplementation (21, 22). Even these scarce studies report controversial findings.

Some of them demonstrated a slight benefit (23), while others failed to show a significant improvement in clinical pregnancy rates (24).

Though VDR null mutant mice are infertile, this condition is corrected by calcium supplementation, suggesting that infertility is due to hypocalcaemia, not to the lack of VD effect on reproductive function (25).

Therefore, this study was aimed at investigating the effect of vitamin D administered at the time of follicle and oocyte development, and at the time of fertilization, on the development and implantation capacity of the embryos from super-ovulated mice.

## Materials and methods

### Mice

CD1 female mice (Charles River, Germany) were housed in an animal airflow cabinet **(**UniProtect NG M, Zoonlab De) controlled for temperature, humidity, and light. The experiments were carried out according to the relevant guidelines and regulations. Experimental protocols were approved by the Animal Health Committee of Baranya County, Hungary.

### Superovulation treatment, retrieval and culture of the embryos

During proestrus, eight to 12 weeks old CD1 female mice (Charles River, Germany) were injected with 5 IU of FSH (IBSA Pharma, Switzerland). Forty-eight hours later, the mice were treated with 5 IU hCG (Choragon, Ferring, Hungary) and directly placed to CD1 males.

Two days after mating, two-cell stage embryos were flushed from the Fallopian tubes, and cultured in 50μl droplets of KSOM medium (Millipore, England), supplemented with 0.4% of BSA, under mineral oil at 37 C°, 5% CO2. Single embryo culture was performed on 96 well plates (Greiner, Germany). Culture media were replaced every 24 hours.

### Calcitriol treatment

Calcitriol, purchased from Cayman Chemical (Ann Arbor, Michigan, USA), was dissolved in ethanol and further diluted in PBS. The mice were treated with an intraperitoneal injection of 100ng Calcitriol in 100 µl PBS.

In Group A, the mice received Calcitriol at the time of the FSH injection. On day 1.5 of pregnancy, embryos were isolated and cultured. Ten mice were sacrificed 2.5 days after Calcitriol treatment for blood collection.

In Group B, Calcitriol was administered at day 0.5 of pregnancy. On day 1.5, embryos were isolated and cultured. Fourteen mice were sacrificed 2.5 days after Calcitriol treatment for blood collection.

Mice in the control group (Group C) underwent the same protocol without Calcitriol injection. Blood was collected from 21 mice corresponding to 2.5 days after the time Calcitriol treatment for both groups.

**Figure 1:**
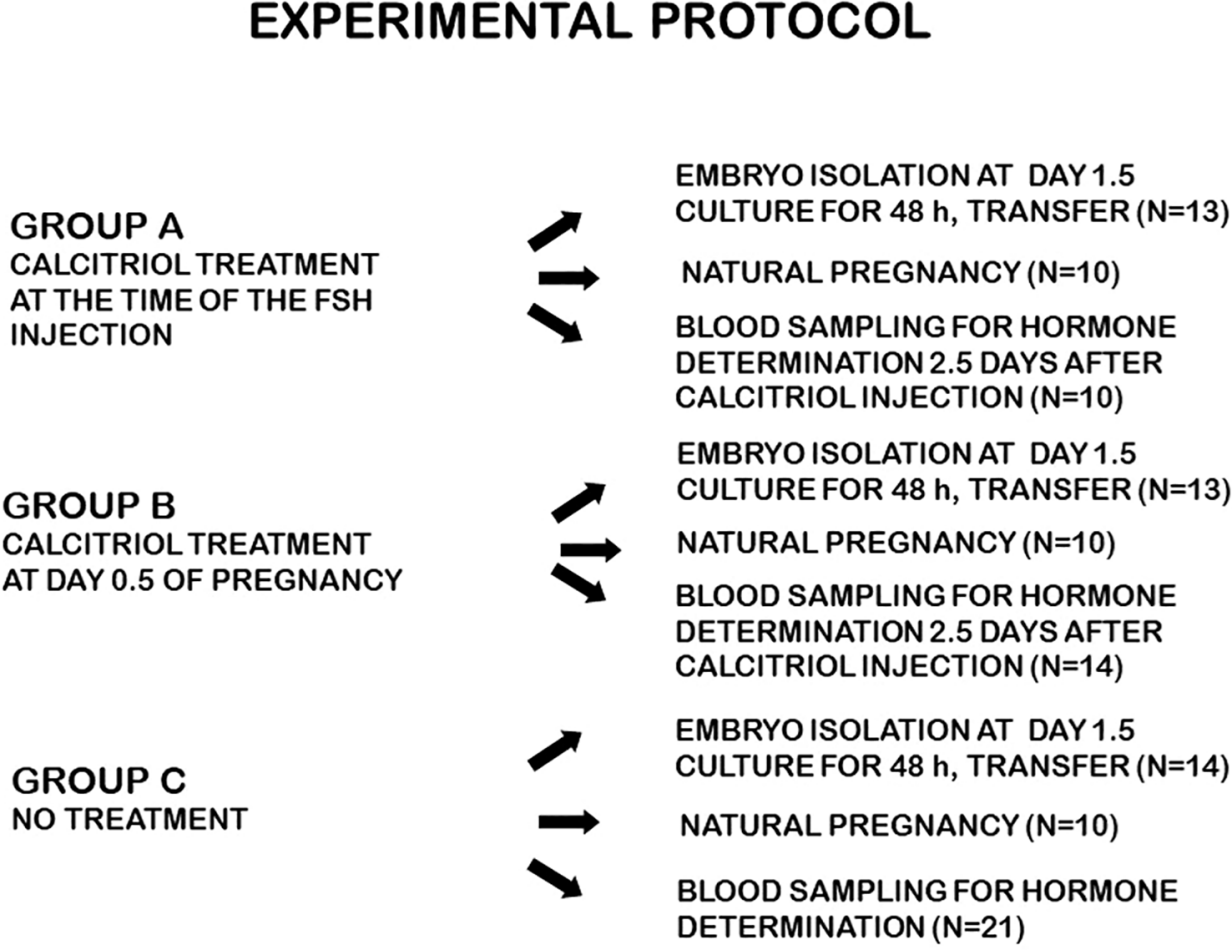
Experimental protocol.

The experimental protocol is shown in **Figure 1**.

### Microscopic evaluation of the developmental potential of the embryos

The embryos were cultured for 2 days, and scored daily for the developmental stage. Those showing signs of degeneration and those, which did not divide during a 24 h culture, were considered non-viable and excluded from the next evaluation. The culture media of the embryos were collected daily, and the samples were frozen for further evaluation.

### Measurement of serum estradiol, progesterone, and 25-hydroxyvitamin D

Serum estradiol and progesterone concentrations were determined by automated competitive chemiluminescent immunoassays (Ref No.: B84493 and 33550) on the Beckman Coulter UniCel DxI 800 Access Immunoassay System (Beckman Coulter, Inc., Brea, CA 92821, U.S.A.). Serum 25-hydroxyvitamin D levels were measured by an automated competitive electrochemiluminescent assay (Ref No.: 09038086190) on the e 801 module of a Cobas 8000 analyzer (Roche Diagnostics GmbH, Mannheim, Germany).

### Histological examination of the ovaries

Paraffin-embedded sections of the ovaries were stained with haematoxylin-eosin.

Ten ovaries from each group were analysed for corpora lutea (CL) and pre-antral follicles (PAF).

### Assessment of the implantation capacity of the embryos

In 10 animals from groups A, B and C, the embryos were not flushed, and pregnancy was allowed to develop until day 7.5, when the mice were sacrificed, and the uteri were scored for the number of implantation sites.

After two days of culture, 106, 101 and 92 embryos from group A, group B and group C (controls), respectively, were transferred to the uteri of (11 for each group) 2.5 dpc pseudo-pregnant females. Five days later the mice were sacrificed and the implantation sites in the uterus were counted.

### Flow cytometric analysis of PIBF content and immune checkpoint ligands expression on extracellular vesicles produced by cultured murine embryos

The expression of CD70, PD-L1, OX-40L, and PIBF was tested by flow cytometry on extracellular vesicles produced by cultured embryos from vitamin D-treated mice.

Measurements were carried out using an Apogee Flow Microflow cytometer (Apogee Flow System, UK), and data were analyzed with the software provided by the manufacturer. The flow cytometer was calibrated with the *ApogeeMix* product (Apogee Flow System, UK). This is a mixture of non-fluorescent silica beads and fluorescent polystyrene beads with sizes from 80nm to 1300nm which can be used to prepare flow cytometers for the analysis of small biological particles by providing points of reference. The extracellular vesicle gate was defined, based on the respective bead sizes. Each sample was measured unstained, and the gate adjusted to the unstained sample, followed by the analysis of the labelled extracellular vesicles within this gate. The results of unconditioned media labelled with the same marker were subtracted from each measurement (**Supplementary Figure S1**).

Twenty μl of embryo culture media were incubated with 1/150 diluted PE anti-mouse CD70 (Clone: FR70, Sony Biotechnology USA), PE anti-mouse CD274 (B7-H1, PD-L1) (Clone: MIH7, Sony Biotechnology USA) and APC anti-mouse CD252 (OX40uligand) (Clone: RM134L, Sony Biotechnology USA) anti-PIBF antibodies (26) for 30 min at room temperature. The binding of the anti-PIBF was visualized by a further 30 min incubation with 1:150 diluted Alexa Fluor 488 goat anti-rabbit IgG secondary antibody (Invitrogen, Thermo-Fisher Scientific, USA).

Unconditioned embryo culture medium was used as the negative control.

### Statistical analysis

The Chi-square-Test was used for comparing the fertilization rates, embryo development, implantation capacity of the embryos, and the rate of CL/PAF. To avoid the decision error, we adjusted the p-values with Bonferroni correction.

The two-tailed t-test was used to analyze the effect of Calcitriol injections on the serum hormones. A p-value under 0.05 was considered significant. All calculations were done with the IBM-SPSS Version 22 software package.

## Results

### Hormone levels in calcitriol-treated mice

Two and a half days after the administration of Calcitriol, serum estradiol concentrations did not significantly differ from those in the controls, while 25(OH) D levels were significantly (p<0.01) decreased in both treated groups. Progesterone concentrations were significantly higher (p<0.01) in group A and lower in group B than in the controls (**Table 1**).

**Table 1:** Progesterone, E2, and 21(OH) D3 values in the sera of superovulated mice, 2.5 days after Calcitriol treatment.

Group A			
	25(OH)D3 (ng/ml)	Estradiol (pmol/l)	Progesterone (nmol/l)
Treated N=10	23.8+/- 1.18^a^	39.5 +/-5.9	37.7+/-4.4^c^
Control N=10	35.1+/- 2.25	44.1 +/- 7.6	21.8+/-3.5
Group B			
	25(OH)D3 (ng/ml)	Estradiol (pmol/l)	Progesterone (nmol/l)
Treated N=14	22.4+/-1.7^b^	28.9 +/-4.3	60.5+/-13^d^
Control N=11	36.2+/-1.3	28.0+/-3.0	96.8+/-9.8

^a^Significantly different from the control at P=0.0008.

^b^Significantly different from the control at P=0.001.

^c^Significantly different from the control at P=0.011.

^d^Significantly different from the control at P=0.048.

### The effect of calcitriol treatment on fertilization and embryo development

**Figure 2:**
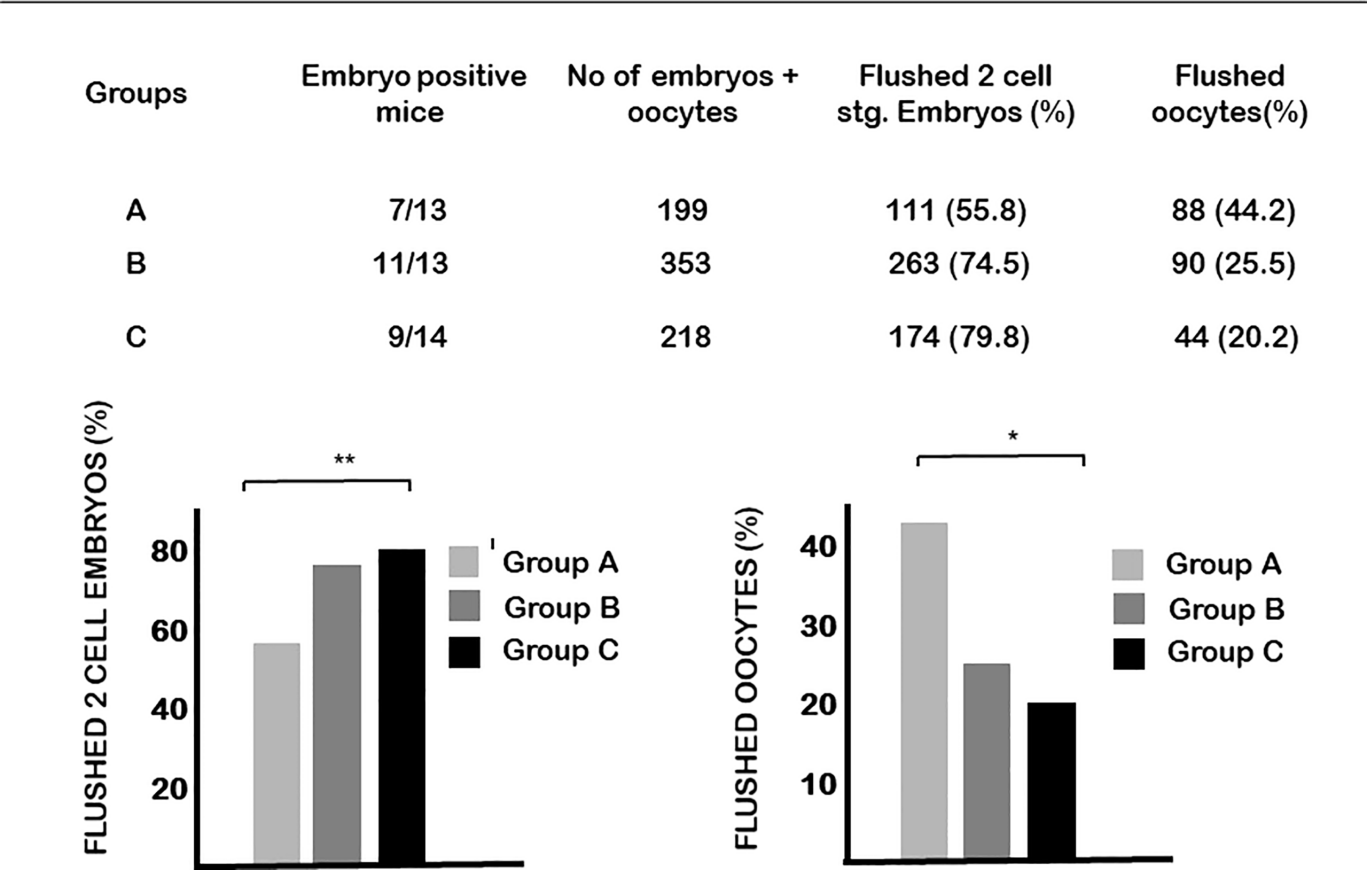
The effect of calcitriol treatment on fertilization. Calcitriol treatment at the time of the FSH injection (group A) significantly decreased the number of two-cell stage embryos obtained on day 1.5 of pregnancy and at the same time increased the ratio of unfertilized oocytes. *p= 0.04, **p=0.01.

Calcitriol treatment at the time of the FSH injection (group A) significantly (p<0.01) decreased the number of two-cell stage embryos obtained on day 1.5 and, at the same time, increased the ratio of unfertilized oocytes (**Figure 2**). The development of cultured embryos was not affected by the treatment.

**Figure 3:**
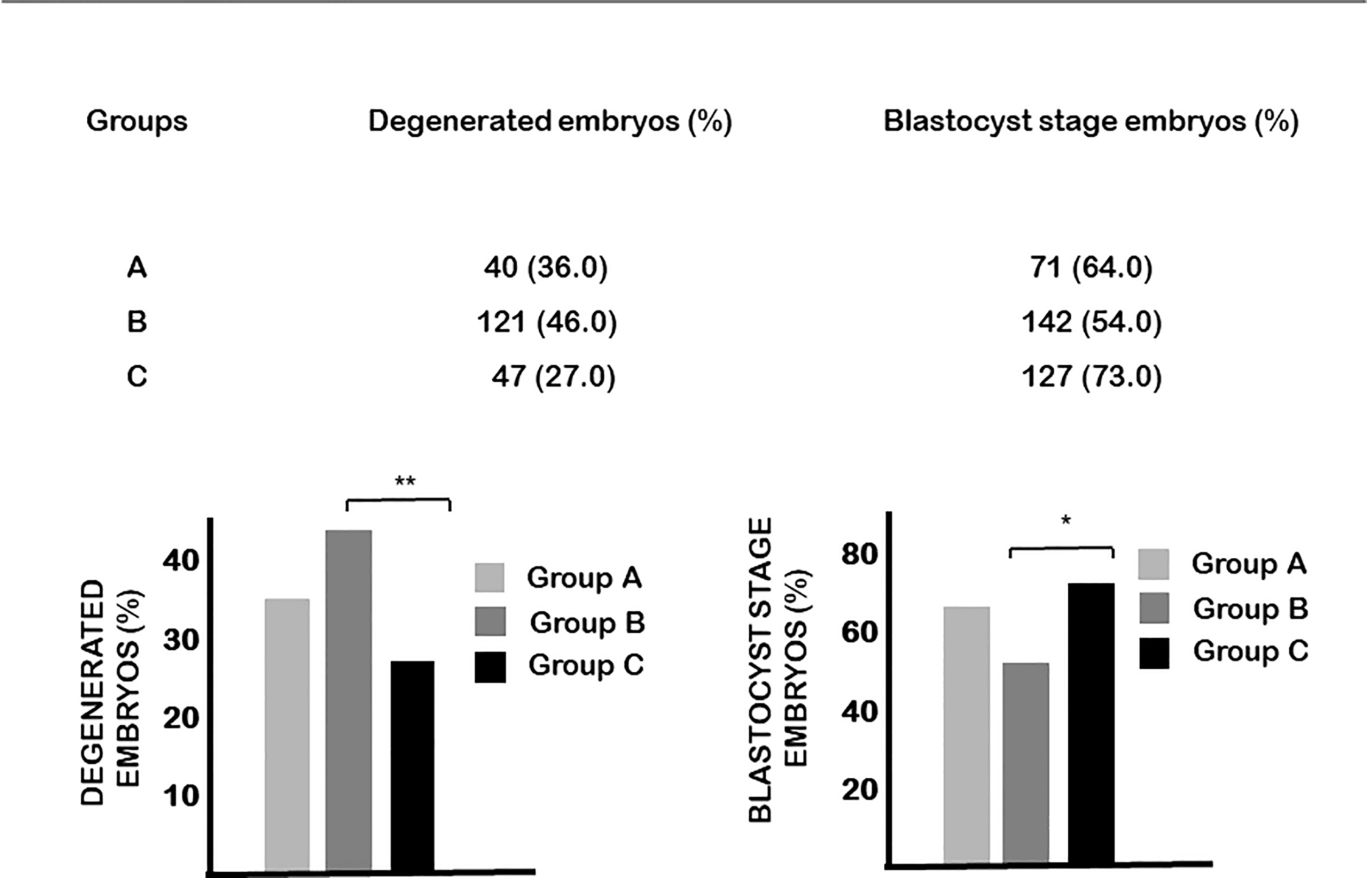
The effect of calcitriol treatment on embryo development. Calcitriol treatment at day 0.5 of pregnancy (group B) significantly decreased the percentage of embryos that developed to blastocyst and increased the percentage of degenerated embryos. *p=0.034, **p=0.004.

On the other hand, the blastulation rate of embryos from mice injected with vitamin D at day 0.5 of pregnancy (group B) was significantly lower, while the rate of degenerated embryos was significantly higher than in the controls (**Figure 3**).

**Figure 4:**
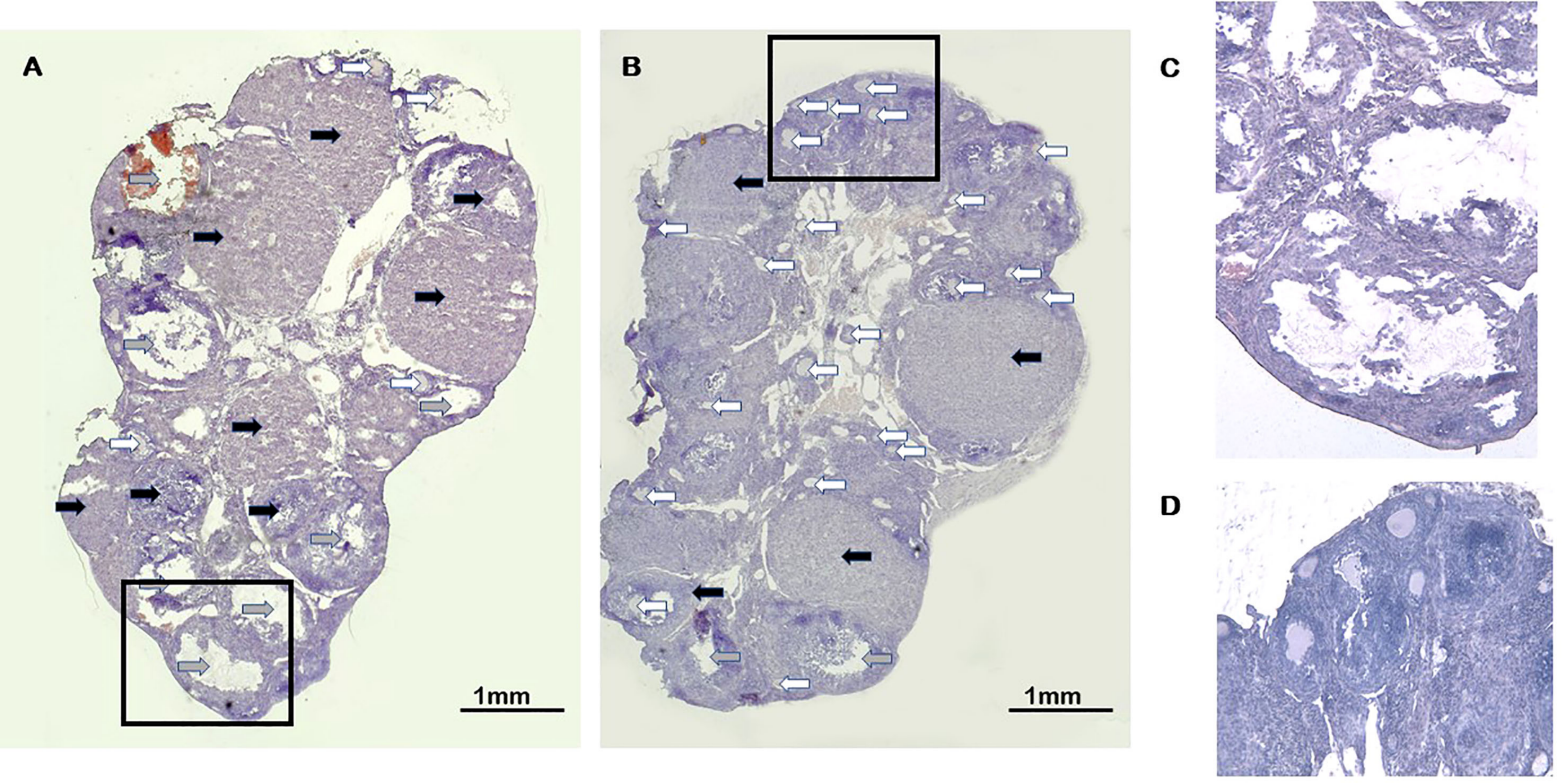
Corpora lutea and pre-antral follicles in ovaries of calcitriol treated mice. **(A)** Control Haematoxylin-eosin staining (20x). **(B)** Calcitriol treatment at the time of FSH injection (group A) Haematoxylin-eosin staining (20x). Black arrows indicate the corpora lutea. Gray arrows show fresh, cystic and haemorrhagic corpora lutea. White arrows point to pre-antral follicles **(C)** High magnification (100x) of corpora luteua. **(D)** High magnification (100x) of pre-antral follicles.

Altogether 74 paraffin-embedded, hematoxylin-eosin-stained sections of the ovaries from group A and 78 from group B mice were scored for corpora lutea (CL) and pre-antral follicles (PAF) (**Figure 4**). In group A mice, we found 5.9+/-0.36 CLs/ovary, significantly (p=0.030) less than in the controls (7.16+/-0.42 CLs/ovary). Simultaneously, the number of pre-antral follicles was increased (8.7+/-0.7/ovary) in the ovaries of group A mice compared to the controls (6.68+/-0.85/ovary), the ratio of CL/PAF being 0.72, compared to the 1.07 found in the ovaries of control animals.

There was no difference in these parameters between group B and control mice.

### Immune checkpoint ligands and PIBF expression on extracellular vesicles produced by Vitamin D–treated and control embryos

Immune checkpoint ligand and PIBF expression of extracellular vesicles produced by morula-blastocyst stage cultured embryos were determined by flow cytometry.

**Figure 5:**
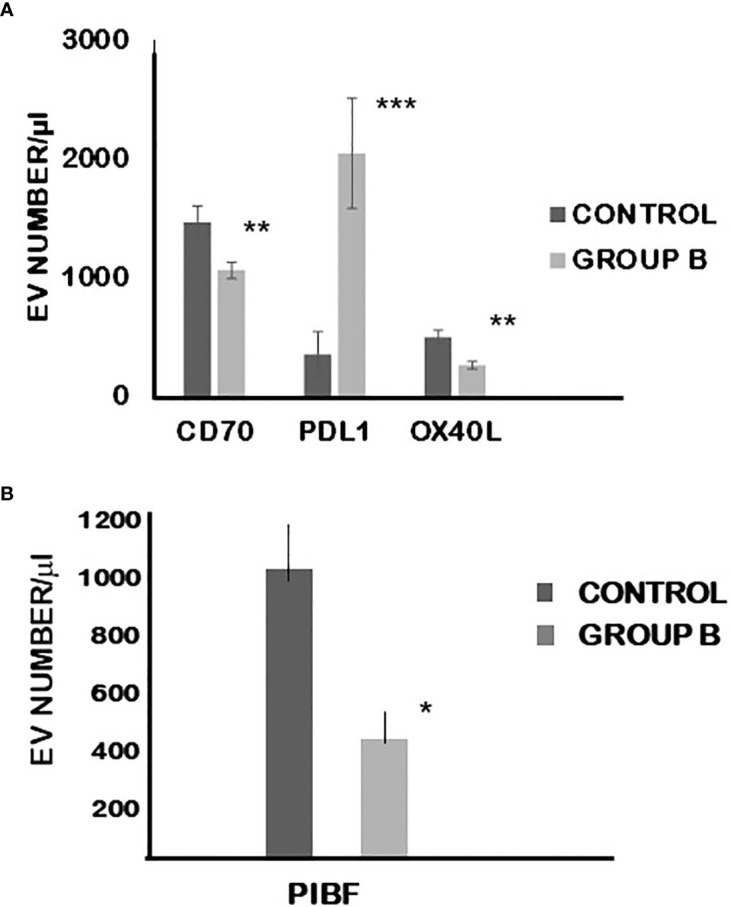
The expression of CD70, PDL-1 and OXL-40 **(A)** as well as PIBF **(B)** on extracellular vesicles produced by cultured embryo from mice treated with calcitriol at day 0.5 of pregnancy. The bars represent the mean+/-SEM of 10 determinations. *p=0.043, **p=0.009 and 0.01, ***p=0.003.

On EVs produced by group B embryos, the expression of PD-L1 was significantly (p< 0.01) higher, while that of CD70, OX-40L (p=0.001) and PIBF was significantly lower (p< 0.05) than on EVs from embryos of control mice (**Figure 5**).

### Implantation capacity of the embryos

One hundred and six, 101 and 92 embryos from group A, group B and group C (controls) were transferred to the uteri of 11, 11 and 10 pseudo-pregnant mice, respectively. On day 7.5 of pregnancy, the mice were sacrificed, and uteri were inspected.

In 10 animals from groups A, B and C, the embryos were not flushed, and pregnancy was allowed to develop until day 7.5, when the mice were sacrificed, and the uteri were scored for the number of implantation sites.

The implantation capacity of both transferred and naturally conceived embryos was similar in the three groups (**Table 2**).

**Table 2:** The effect of Calcitriol treatment on the implantation capacity of embryos.

Groups	Group A	Group B	Group C
Embryo transfer			
No. of mice	11	11	11
Embryos transferred	106	101	92
Embryos implanted	47	56	51
Rate of implantation	44.34%	55.40%	55.43%
Significance	NS	NS	NS
Natural mating			
No. of mice	10	10	10
Pregnant mice	6	5	4
Average of implanted embryos	13.3	15.8	15.4
Difference between the groups	NS	NS	NS

## Discussion

The duration of the implantation window is regulated by estradiol (27). Treatment with 1,25(OH)2 D3 has been shown to significantly decrease the levels of ER-α, PR-A, and PR-B, in immortalized human uterine cells (21). In an *in vivo* study 1,25-dihydroxyvitamin D3 treatment of female rats with uterine leiomyomas reduced the expression of oestrogen and progesterone receptors in the tumour tissue (22).

These studies suggest, that 1,25 (OH)2D3 negatively regulates the expression of sex steroid receptors.

Though serum oestrogen levels of calcitriol-treated mice did not differ from those of the controls, based on the above data (21, 22), it cannot be ruled out, that Calcitriol treatment might have downregulated the expression of oestrogen and progesterone receptors. This would explain the unfavourable effect of 1,25(OH)2D3 on follicular development (lower number of fresh corpora lutea together with high number of pre-antral follicles) and the lower fertilization rate.

Calcitriol treatment during the pro-oestrus (group A) significantly increased serum progesterone concentrations. This might be a sign of pre-ovulatory progesterone rise, which by negatively affecting embryo quality and implantation potential, has an adverse effect on pregnancy rates (28).

Calcitriol treatment at day 0.5 of pregnancy (group B) decreased the progesterone levels compared to the controls. This resulted in impaired development of the embryos, characterized by the high number of degenerated embryos and the low number of embryos that reached the blastocyst stage during culture.

The success of implantation and pregnancy depends on a favourable immunological environment. Concerted action of progesterone and vitamin D regulate T cell function. Progesterone induces vitamin D receptors in T cells and thus makes T cells highly sensitive to calcitriol, even at low vitamin D concentrations. This regulatory pathway allows enhanced induction of Tregs but suppression of Th1 and Th17 cells by the two nuclear hormones (29).. Treatment of ectopic endometrial cells with 1,25 (OH)_2_D_3_ significantly reduced cytokine-mediated inflammatory responses (29).

Via its downstream mediator, PIBF, progesterone plays a role in the fine-tuning of the immune milieu. The embryo, and later the foetus, communicates with the maternal immune system by messages sent via extracellular vesicles. Extracellular vesicles produced by the cultured embryo contain PIBF and when reaching the maternal side, induce increased production of Th2-type cytokines (30), which is required for normal implantation (31).

Here we show that the number of PIBF+ EVs produced by embryos of calcitriol-treated mice is reduced, compared to that produced by the control embryos. Furthermore, the expression of certain immune-checkpoint ligands was altered in EVs produced by embryos of calcitriol-treated mice.

The expression of PD-L1 was significantly higher, while that of CD70, and OX-40L were significantly lower than on EVs produced by embryos of control mice. Engagement of PD-L1 with its receptor PD-1 on T cells delivers a signal that inhibits T cell receptor-mediated activation of IL-2 production and T cell proliferation. CD 70 is the ligand of the CD27 molecule expressed on T cells and NK cells. The CD70/CD27 pathway influences the polarization of CD4^+^ T cells towards Th1. OX40 is a member of the TNF receptor family and plays a role in the survival of effector and memory T cells. OX40–OX40L interaction regulates T-cell tolerance and peripheral T-cell homeostasis. Altered expression patterns of the different immune checkpoint ligands in D vitamin-treated mice could interfere with the maternal immunomodulatory mechanisms favouring healthy pregnancy.

In summary, our findings revealed an unfavourable effect of VD supplementation on oocyte and embryo development in mice, with no impact on the implantation rates. Calcitriol treatment at 0.5 day of pregnancy resulted in decreased serum progesterone concentrations. This, together with the possible negative effect of vitamin D on progesterone receptor expression (21, 22), might have led to impaired PIBF production of the embryos. On the other hand, the altered expression of immune checkpoint molecules and ligands on embryo-derived EVs suggests a favourable message to the maternal immune system.

Our findings are in line with the data reported in a recent meta-analysis of five randomized controlled trials (32), showing that vitamin D supplementation was associated with improved chemical pregnancy rate, but did not improve the fertilization rate, the number of good quality embryos or clinical pregnancy rate.

We have to point out that in this study, we concentrated on the embryo and have yet to investigate the effect of VD on endometrial receptivity. However, the fact that the implantation rates of embryos from calcitriol-treated mice did not differ from those of the controls suggests that the beneficial effect of vitamin D on pregnancy outcome might not be attributed to the effect of VD on reproductive function.

## Data availability statement

The raw data supporting the conclusions of this article will be made available by the authors, without undue reservation.

## Ethics statement

The animal study was reviewed and approved by Institutional animal experiment ethics committee.

## Author contributions

ZB and JSB designed the study. ZB, TC-T, EG, EM and ZH-S. performed the research. JSB, ZB and EM discussed and analyzed the data. ZB and JSB wrote the paper. JSB revised the manuscript. All authors contributed to the article and approved the submitted version.

## Supplementary material

Supplementary Figure 1 Calibration and gating strategy for detecting PIBF and immune checkpoint ligand on embryo-derived extracellular vesicles. The flow cytometer was calibrated with the *ApogeeMix* (Hemel Hempstead, Hertfordshire, UK) which is a mixture of non-fluorescent silica beads and fluorescent polystyrene beads with sizes from 80nm to 1300nm.This can be used to prepare flow cytometers for the analysis of small biological particles by providing points of reference. **(A)** The extracellular vesicle gates were defined based on the bead sizes (110-880 nm) **(B)**. Each sample was measured unstained, and the quadrant was adjusted to the unstained sample **(C)**. The labelled extracellular vesicles were analysed within this gate **(D)**. The results of unconditioned media labelled with the same marker were subtracted from each measurement.
