# Supplementary figures and images for: The effect of calcitriol on the development and implantation capacity of embryos from hyper-stimulated mice
Source: Front Immunol. 2023 Jul 21;14:1200704. doi: 10.3389/fimmu.2023.1200704 (PMC10401042; doi:10.3389/fimmu.2023.1200704)

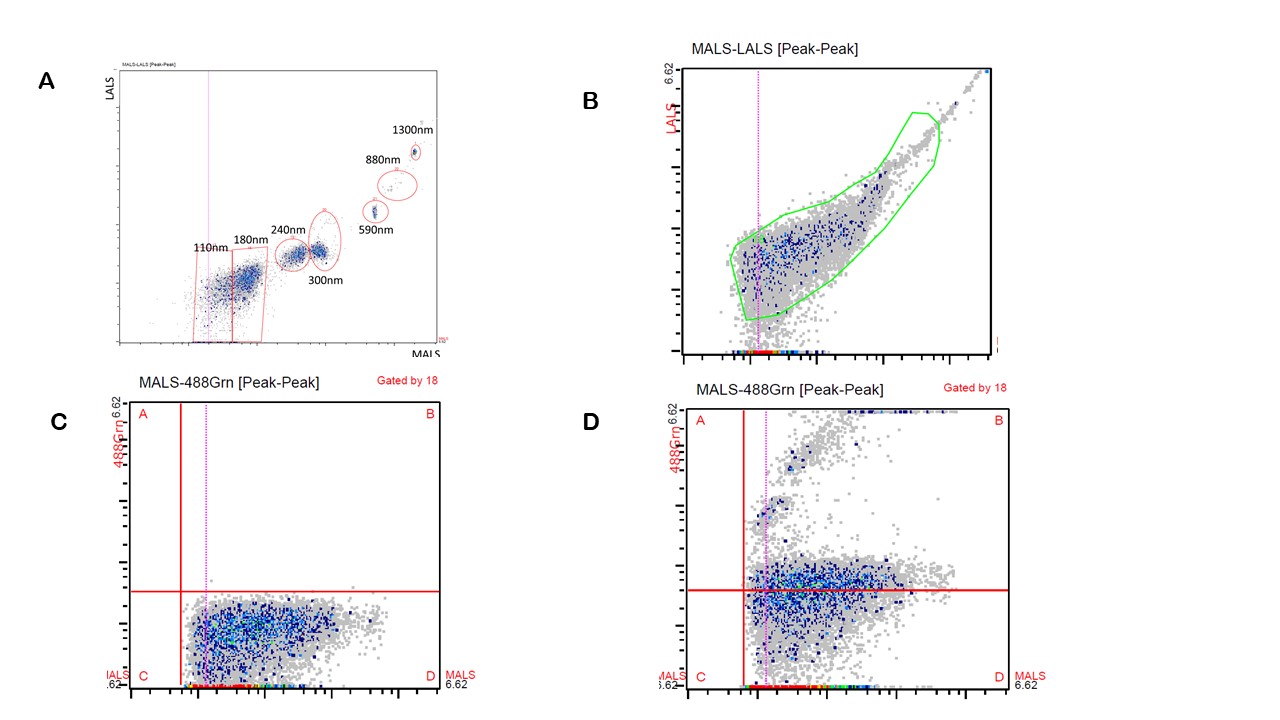

Supplement: Supplementary Figure 1 — Calibration and gating strategy for detecting PIBF and immune checkpoint ligand on embryo-derived extracellular vesicles. The flow cytometer was calibrated with the ApogeeMix (Hemel Hempstead, Hertfordshire, UK) which is a mixture of non-fluorescent silica beads and fluorescent polystyrene beads with sizes from 80nm to 1300nm.This can be used to prepare flow cytometers for the analysis of small biological particles by providing points of reference. (A) The extracellular vesicle gates were defined based on the bead sizes (110-880 nm) (B). Each sample was measured unstained, and the quadrant was adjusted to the unstained sample (C). The labelled extracellular vesicles were analysed within this gate (D). The results of unconditioned media labelled with the same marker were subtracted from each measurement. [file Image_1.jpeg]
